# Thromboelastometry-guided hemostatic therapy: an efficacious approach to manage bleeding risk in acute fatty liver of pregnancy: a case report

**DOI:** 10.1186/s13256-015-0690-9

**Published:** 2015-09-23

**Authors:** Tomaz Crochemore, Felipe Maia de Toledo Piza, Eliézer Silva, Thiago Domingos Corrêa

**Affiliations:** Intensive Care Unit, Hospital Israelita Albert Einstein, Av. Albert Einstein, 627, São Paulo, SP CEP: 05651-901 Brazil

## Abstract

**Introduction:**

Acute fatty liver of pregnancy (AFLP) is a rare but life-threatening disease. AFLP is characterized by liver failure with different degrees of coagulopathy. Outcome and survival can be dramatically improved with prompt recognition and treatment. Thromboelastometry has been considered a point of care for the management of bleeding patients. It could, therefore, be an alternative tool to treat the complex cases of AFLP involving liver failure and coagulopathy. Through this study, we present our successful experience of an AFLP case that was submitted to an emergency cesarean section in which blood transfusion was guided by thromboelastometry.

**Case presentation:**

We report the case of a previously healthy 28-year-old woman, Afro-Brazilian, in her first pregnancy with no medical records until the 36^th^ pregnancy week. She presented to our emergency department with an acute onset of abdominal pain, jaundice, nausea and vomiting. The laboratory examinations revealed metabolic acidosis, acute kidney injury (serum creatinine 3.4mg/dL), platelets 97 × 10^3^/mm^3^, serum fibrinogen 98mg/dL and increased international nationalized ratio (INR 6.9) without acute bleeding. An emergency cesarean section was indicated. Based on the results of the thromboelastometric tests EXTEM and FIBTEM, prothrombin complex concentrate and fibrinogen concentrate were administered at the beginning of the cesarean section, which succeeded with no major bleeding and without need of further transfusion.

**Conclusions:**

Thromboelastometry may be considered a useful, feasible and safe tool to monitor and manage coagulopathy in obstetric patients with acute fatty liver of pregnancy, with the potential advantage of helping avoid unnecessary transfusion in such patients.

## Introduction

Acute fatty liver of pregnancy (AFLP) represents a rare but potentially fatal pregnancy complication, usually diagnosed during the last trimester or early in the postpartum period [1]. The clinical picture of AFLP is nonspecific and comprises headache, fatigue, anorexia, nausea, vomiting, abdominal pain, fever and jaundice [1]. However, the most severe spectrum of the AFLP is characterized by an early multiorgan involvement [1]. Liver failure is the landmark of the AFLP and may be accompanied by encephalopathy, gastrointestinal bleeding, acute kidney injury and different degrees of coagulopathy, which intensify the risk of obstetric hemorrhage and death [2].

The histopathological characteristic of AFLP is disseminated microvesicular fatty infiltration of hepatocytes with minimal cholestasis without necrosis [3]. The AFLP affects 1 in 7,000 to 13,000 pregnancies [3]. Maternal mortality rate has been estimated to be as high as 18%, and the neonatal mortality rate can range from 7% up to 58% [4]. The pathophysiology of AFLP is not completely understood but this condition has been associated with an inherited deficiency of the mitochondrial enzyme long-chain 3-hydroxyacetyl-coenzyme-A dehydrogenase (LCHAD) [1].

It has been postulated that HELLP syndrome (hemolysis, elevated liver enzymes and low platelets), preeclampsia, thrombotic thrombocytopenic purpura and AFLP may all be a spectrum of the same disease [4, 5]. Therefore, its early diagnosis represents a challenge for the clinicians at the bedside [4]. Most patients will recover within 4 weeks after the delivery [6]. Nevertheless, it is important to emphasize that outcomes and survival can be dramatically improved with timely recognition and prompt treatment [6].

Thromboelastometry represents a viscoelastic test that has been considered an important tool for the management of critically ill patients [7]. It has been used mainly for early prediction of bleeding complications and goal-directed therapy in different scenarios, such as trauma, septic shock, anesthesia, hepatic and cardiac surgeries [8]. It is well known that standard plasmatic coagulation screening tests such as activated partial thromboplastin time (aPTT) or prothrombin time (PT) represent weak predictors of bleeding in critically ill patients; they represent suboptimal tests for monitoring coagulopathy and guide hemostatic therapy [7].

Therefore, thromboelastometry-guided hemostatic therapy could be considered an alternative tool to manage complex cases of AFLP that are characterized by liver failure and coagulopathy. We are not aware of any report addressing the thromboelastometry role to monitor the coagulation system and guide hemostatic therapy in AFLP patients. Consequently, our objective was to describe a case of an acute fatty liver of a pregnant patient in which thromboelastometry was successfully used to identify coagulopathy and guide transfusion.

## Case presentation

We report the case of a previously healthy 28-year-old woman, Afro-Brazilian, in her first pregnancy. Our patient had no medical records until the 36^th^ pregnancy week and reported allergy to diclofenac. She presented to our emergency department with an acute onset of abdominal pain, jaundice, nausea and vomiting, with no signs of encephalopathy. Her arterial blood pressure was 110/60mmHg, heart rate was 98bpm, axillary temperature was 35°C, she was severely dehydrated and with decreased peripheral perfusion.

The laboratory examinations revealed hemoglobin 12.3g/dL, leukocytes 13 × 10^9^/mL, platelets 97 × 10^3^/mm^3^, international nationalized ratio (INR) 6.9, fibrinogen 98mg/dL, total bilirubin 14.2mg/dL, serum creatinine 3.4mg/dL, serum aspartate aminotransferase (AST) 306U/L, serum alanine aminotransferase (ALP) 302U/L, arterial bicarbonate 11mEq/L, arterial pH 7.21, blood glucose 65mg/dL and ionic calcium 1.02mmol/L. An abdominal ultrasound depicted fatty infiltration of the liver and confirmed fetal viability.

**Fig. 1:**
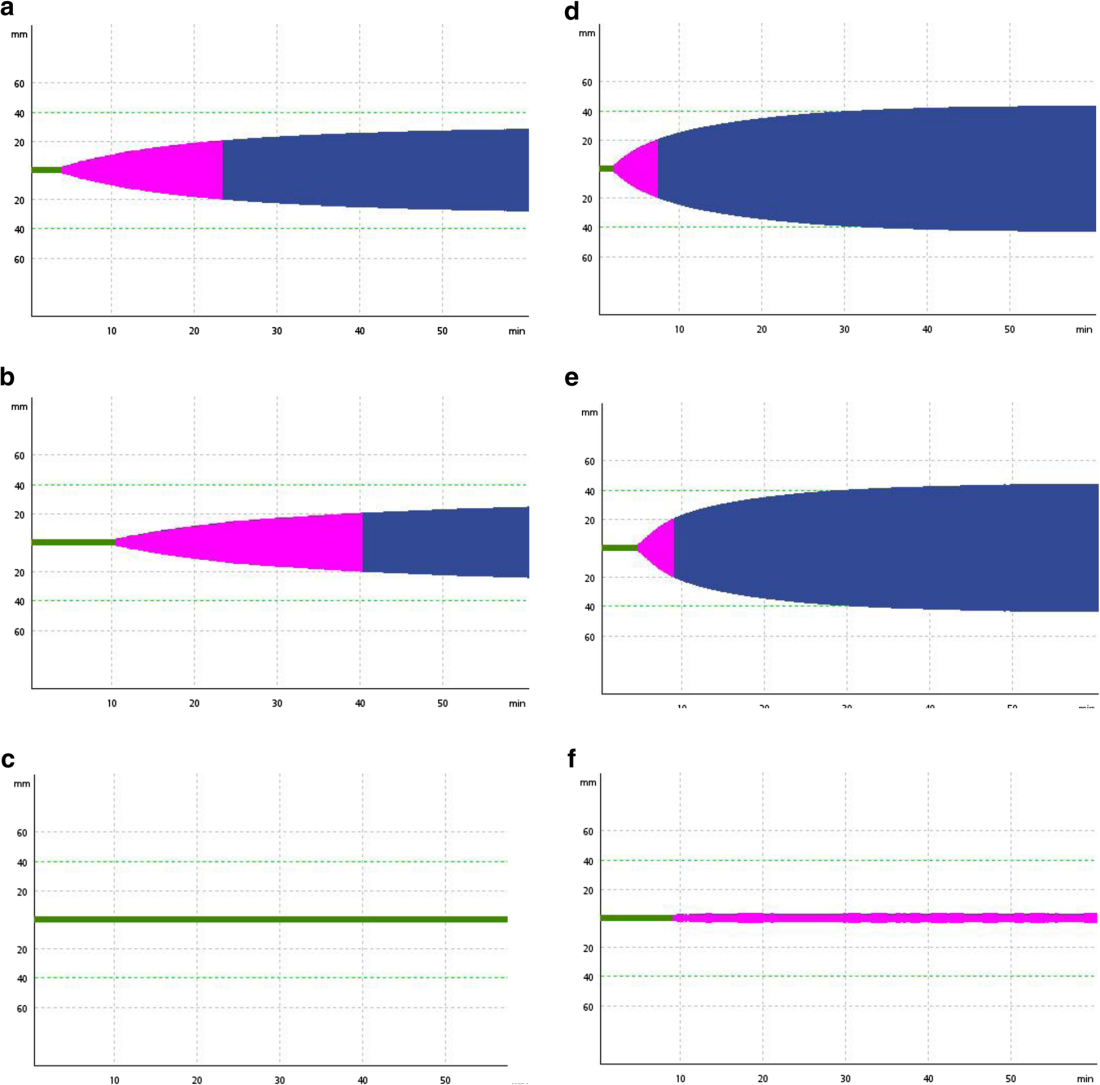
Thromboelastometry analysis (ROTEM®) at the beginning of the cesarean section. **a-c** Thromboelastometry analysis (ROTEM®) performed at the beginning of the cesarean section (time 0) showing an intense kinetic and structural hypocoagulable state. **a** EXTEM showing a coagulation factor deficiency. **c** FIBTEM showing an impairment in fibrinogen function quality. **d-f** Second thromboelastometry analysis performed after 4.0g of fibrinogen concentrate (Haemocomplettan® P, CSL Behring, Marburg, Germany) and 1000UI of prothrombin complex concentrate (Beriplex® P/N 500UI, CSL Behring, Marburg, Germany) administration (time 3 hours)

Our patient received an initial fluid load with crystalloids. The calcium, glucose and hypothermia were reversed. The diagnosis of AFLP was confirmed following the Swansea’s criteria [9]. Therefore, a cesarean section was indicated. A thromboelastometry (ROTEM®, Pentapharm Co., Munich, Germany) was performed at the beginning of the surgery. The thromboelastometry analysis showed an intense kinetic and structural hypocoagulable state (Fig. 1 and Table 1). The FIBTEM revealed an impairment in fibrinogen function quality while the EXTEM depicted a coagulation factor deficiency (Fig. 1a-c and Table 1).

**Table 1:** Sequential thromboelastometry (ROTEM®) analysis from the emergency department admission through intensive care unit discharge

Time (hours)	Assays	CT (s)	CFT (s)	α angle (°)	MCF (mm)	LI60 (%)
0	EXTEM	228	1168	22	29	100
	INTEM	614	1792	16	27	100
	FIBTEM	-	-	-	0	-
3	EXTEM	109	328	50	43	100
	INTEM	213	302	50	43	100
	FIBTEM	-	-	-	3	-
24	EXTEM	70	318	43	44	100
	INTEM	265	265	50	44	100
	FIBTEM	-	-	-	5	-
36	EXTEM	62	282	58	42	100
	INTEM	224	228	57	43	100
	FIBTEM	-	-	-	10	-

*CT* clotting time, *CFT* clot formation time, *MCF* maximum clot firmness, *LI60* lysis index 60

Based on, respectively, FIBTEM maximum clot firmness (MCF) (0mm; Table 1) and EXTEM clotting time (CT) (228s; Table 1), 4.0g of fibrinogen concentrate (Haemocomplettan® P, CSL Behring, Marburg, Germany) and 1000UI of prothrombin complex concentrate (Beriplex® P/N 500UI, CSL Behring, Marburg, Germany) were administered at the beginning of the cesarean section. The fluid input (crystalloids) and output during the caesarian section were, respectively, 2000mL and 200mL. The cesarean section succeeded with no major bleeding after the hemostatic therapy.

Additional hemocomponent transfusion, such as fresh frozen plasma (FFP), cryoprecipitate, platelets or blood concentrates, was not necessary. A second thromboelastometry analysis was performed at the end of surgery (Fig. 1d-e and Table 1), showing a mild hypocoagulation state. The patient was admitted to the intensive care unit (ICU) and remained stable, with no bleeding during the recovery phase. She was discharged from the ICU 3 days after admission and then 3 days later she was discharged from the hospital.

## Discussion

Acute fatty liver of pregnancy occurs suddenly during the last trimester, when fatty infiltration of hepatocytes will cause hepatic failure and consequently encephalopathy and different degrees of coagulopathy [5]. Although its exact pathogenesis is not completely known, this disease has been associated with an abnormality in fetal fatty acid metabolism [3]. AFLP can lead to significant maternal and fetal morbidity and mortality. In the past, this condition was practically fatal. However, nowadays it is possible to stabilize the condition of the mother with safety through the available intensive care resources.

The diagnosis of AFLP is still a challenging task for clinicians because of its nonspecific clinical presentation, which may be mistaken with other diseases as such HELLP syndrome, acute viral hepatitis and preeclampsia [5]. The clinical findings of AFLP are characterized by varying degrees of severity along with the classical symptoms of the third pregnancy trimester, making early diagnosis difficult [2]. Patients usually show nonspecific symptoms such as anorexia, nausea, vomiting, malaise, fatigue, headache and abdominal pain [2]. In the most severe cases, patients may have dysfunctions of additional systems, including acute renal failure, hepatic encephalopathy, pancreatitis, gastrointestinal bleeding and coagulopathy [10].

Laboratory abnormalities detected with conventional coagulation tests such as aPTT, PT, fibrinogen and platelet count can be prophylactically corrected with replacement of FFP, cryoprecipitate, packed red cells or platelet concentrates [11]. Nevertheless, the current literature suggests that prophylactic transfusion of blood products aiming to correct laboratory abnormalities increases the risk of acute lung injury, fluid overload, infection, immunosuppression, organ dysfunction and, therefore, should not be performed [12]. Furthermore, it is well known that the standard plasmatic coagulation screening tests represent weak predictors of bleeding in the critically ill patients and represent suboptimal tests for monitoring coagulopathy or guiding hemostatic therapy [13].

The principle of thrombelastography was described in 1948. Thrombelastography is a method, unlike the conventional or standard coagulation tests, which is able to monitor the whole-blood coagulation system and determine the clot strength as well as the lytic processes [14]. By providing a graphic presentation of the fibrin polymerization and lysis, thrombelastography reflects the viscoelastic changes that occur during the coagulation process [14]. Although thrombelastography provides interesting analytical information from the very beginning of clot formation, it was seldom used in routine clinical practice because of its extreme sensitivity to vibration and mechanical shock.

However, at the beginning of the 1990s, the principle of thromboelastometry (ROTEM®) was developed. In contrast to the classical thrombelastography, thromboelastometry is practically insensitive to environmental artefacts and it is easy to handle. In contrast to the original method, automated pipetting in ROTEM allows complex coagulation analyses to be performed.

As pointed out before, thromboelastometry represents a viscoelastic test that has been considered an important tool for the management of critically ill patients [7]. Thromboelastometry enables a contemporary detection of several hemostatic disorders within 5 minutes (amplitude at 5 minutes) after CT EXTEM, INTEM and FIBTEM in patients with severe coagulopathy [15, 16]. It has been used mainly for early prediction of bleeding complications and goal-oriented therapy with specific hemostatic drugs such as coagulation factor concentrates and blood products in different patients, including trauma, sepsis, anesthesia, liver and cardiac surgeries.

The ROTEM® allows four independent measuring channels and assays. Several activators and additives are commercially available nowadays (EXTEM, INTEM, FIBTEM, HEPTEM and APTEM) and are used to detect specific hemostatic defects such as coagulation factor deficiencies, thrombocytopenia, heparin and protamine effects, hypofibrinogenemia, hyperfibrinolysis and finally, fibrin polymerization disorders.

For the interpretation of thromboelastometric assays, the following variables should be analyzed together: CT, clot formation time (CFT) and MCF. Clotting time represents the time from adding the start reagent to the citrated blood sample until the clot starts to form (clot firmness of 2mm). Prolongation of the CT may be the result of coagulation factor deficiencies or the effect of anticoagulants such as heparin. Furthermore, it has been demonstrated that CT prolongation may also occur when not enough fibrinogen is available for clot formation [17–19]. Therefore, a stepwise approach for hemostatic therapy is advisable. When CT is prolonged, fibrinogen should be replaced first and then, if CT remains prolonged, coagulation factor deficiency should be corrected with FFP or prothrombin complex concentrate administration.

An abnormal clot formation is indicated by a prolonged clot formation time (period from 2mm to 20mm of amplitude) and/or a reduced maximum clot firmness. The maximum clot firmness represents the greatest amplitude of the thromboelastometric trace and reflects the “strength” of the clot. A low MCF is indicative of decreased platelet concentration and/or function, decreased fibrinogen concentration and/or fibrin polymerization disorders, or low activity of factor XIII. A mechanically weak clot represents a severe bleeding risk. Fibrinolysis is detected by the lysis of the clot [maximum lysis (ML) > 15%].

The initial results of conventional coagulation tests of the previously presented case revealed a low platelet count and fibrinogen and an increased INR. Based on these results, our patient should have received FFP, platelets and cryoprecipitate to avoid peri-operative bleeding. Nevertheless, the ROTEM® depicted a severe kinetic and structural hypocoagulable state secondary to hypofibrinogenemia and coagulation factor deficiency. These results led us to an early identification of structural coagulopathy and the underlying coagulation disorder. By administering fibrinogen concentrates and prothrombin complex, it was possible to correct the coagulation disorder avoiding intraoperative bleeding. More importantly, with no need of blood products administration.

It is important to emphasize that conventional coagulation tests do not allow us to address the underlying coagulation disorder, often resulting in a replacement of blood components unnecessarily [12]. The thromboelastometry analysis allows clinicians to recognize the presence of coagulopathy and, most importantly, to understand the underlying clotting disorder. Early ROTEM-guided transfusion in the case presented avoided unnecessary blood transfusions, minimizing the risk of serious transfusion-related complications, such as acute lung injury (TRALI), transfusion acute cardiac overload (TACO) and risk of infections [20].

Our patient had a serious case of hypofibrinogenemia and deficiency of coagulation factors. Observational studies have been suggesting an association between low fibrinogen levels and the risk and severity of postpartum hemorrhage [21]. Therefore, the increased bleeding risk in this population of patients might overcome the potential risk of adverse reactions associated with fibrinogen concentrate administration. More importantly, it was possible to correct the hypofibrinogenemia by replacing 4.0g of fibrinogen concentrate. Had we used conventional tests to guide blood transfusion therapy, we would have used FFP and cryoprecipitate instead of fibrinogen concentrate and prothrombin complex concentrate, thereby increasing the risk of blood-related adverse events. Additionally, it is well known that FFP contains low amounts of fibrinogen, approximately 250mg per unit. Therefore, 16 units of FFP would be necessary to replace the same 4.0g of fibrinogen administered with fibrinogen concentrate.

## Conclusions

In summary, successful management of patients with AFLP is a challenge. Both outcome and survival rate can be improved with prompt recognition and early treatment. Thromboelastometry may be considered a useful, feasible and safe tool to monitor and manage coagulopathy in obstetric patients with AFLP. Nevertheless, additional studies are needed to define the actual benefit of thromboelastometry for early coagulopathy identification, bleeding predictor, and finally as a guide for blood products and hemostatic drugs.

## Consent

Written informed consent was obtained from the patient for publication of this case report and any accompanying images. A copy of the written consent is available for review by the Editor-in-Chief of this journal.

## Abbreviations

AFLP: acute fatty liver of pregnancy
ALP: serum alanine aminotransferase
aPTT: activated thromboplastin time
AST: serum aspartate aminotransferase
CFT: clot formation time
CT: clotting time
FFP: fresh frozen plasma
ICU: intensive care unit
HELLP: hemolysis, elevated liver enzymes and low platelet count
INR: international nationalized ratio
LCHAD: long-chain 3-hydroxyacetyl-coenzyme-A dehydrogenase
LI60: lysis index 60
MCF: maximum clot firmness
ML: maximum lysis
PT: prothrombin time
ROTEM: thromboelastometry
TACO: transfusion acute cardiac overload
TRALI: transfusion-related acute lung injury

## References

[1] Kaplan MM. Acute fatty liver of pregnancy. N Engl J Med. 1985;313:367–70. 10.1056/NEJM198508083130606 3892299

[2] Fesenmeier MF, Coppage KH, Lambers DS, Barton JR, Sibai BM. Acute fatty liver of pregnancy in 3 tertiary care centers. Am J Obstet Gynecol. 2005;192:1416–9. 10.1016/j.ajog.2004.12.035 15902124

[3] Ibdah JA. Acute fatty liver of pregnancy: an update on pathogenesis and clinical implications. World J Gastroenterol. 2006;12:7397–404. 10.3748/wjg.v12.i46.7397 PMC4087582 17167825

[4] Bacq Y. Liver diseases unique to pregnancy: a 2010 update. Clin Res Hepatol Gastroenterol. 2011;35:182–93. 10.1016/j.clinre.2010.11.011 21310683

[5] Ko H, Yoshida EM. Acute fatty liver of pregnancy. Can J Gastroenterol. 2006;20:25–30. 10.1155/2006/638131 PMC2538964 16432556

[6] Knight M, Nelson-Piercy C, Kurinczuk JJ, Spark P, Brocklehurst P. A prospective national study of acute fatty liver of pregnancy in the UK. Gut. 2008;57:951–6. 10.1136/gut.2008.148676 18332072

[7] Meybohm P, Zacharowski K, Weber CF. Point-of-care coagulation management in intensive care medicine. Crit Care. 2013;17:218. 10.1186/cc12527 PMC3672634 23510484

[8] Pezold M, Moore EE, Wohlauer M, Sauaia A, Gonzalez E, Banerjee A, et al. Viscoelastic clot strength predicts coagulation-related mortality within 15 minutes. Surgery. 2012;151:48–54. 10.1016/j.surg.2011.06.023 PMC4364997 21899867

[9] Goel A, Ramakrishna B, Zachariah U, Ramachandran J, Eapen CE, Kurian G, et al. How accurate are the Swansea criteria to diagnose acute fatty liver of pregnancy in predicting hepatic microvesicular steatosis? Gut. 2011;60:138–9. 10.1136/gut.2009.198465 20938054

[10] Moldenhauer JS, O’Brien JM, Barton JR, Sibai B. Acute fatty liver of pregnancy associated with pancreatitis: a life-threatening complication. Am J Obstet Gynecol. 2004;190:502–5. 10.1016/j.ajog.2003.09.022 14981397

[11] Thomas D, Wee M, Clyburn P, Walker I, Brohi K, Collins P, et al. Blood transfusion and the anaesthetist: management of massive haemorrhage. Anaesthesia. 2010;65:1153–61. 10.1111/j.1365-2044.2010.06538.x PMC3032944 20963925

[12] Stainsby D, Jones H, Asher D, Atterbury C, Boncinelli A, Brant L, et al. Serious hazards of transfusion: a decade of hemovigilance in the UK. Transfus Med Rev. 2006;20:273–82. 10.1016/j.tmrv.2006.05.002 17008165

[13] Chowdary P, Saayman AG, Paulus U, Findlay GP, Collins PW. Efficacy of standard dose and 30ml/kg fresh frozen plasma in correcting laboratory parameters of haemostasis in critically ill patients. Br J Haematol. 2004;125:69–73. 10.1111/j.1365-2141.2004.04868.x 15015971

[14] Johansson PI, Stissing T, Bochsen L, Ostrowski SR. Thrombelastography and tromboelastometry in assessing coagulopathy in trauma. Scand J Trauma Resusc Emerg Med. 2009;17:45. 10.1186/1757-7241-17-45 PMC2758824 19775458

[15] Song JG, Jeong SM, Jun IG, Lee HM, Hwang GS. Five-minute parameter of thromboelastometry is sufficient to detect thrombocytopenia and hypofibrinogenaemia in patients undergoing liver transplantation. Br J Anaesth. 2014;112:290–7. 10.1093/bja/aet325 24065728

[16] Haas T, Gorlinger K, Grassetto A, Agostini V, Simioni P, Nardi G, et al. Thromboelastometry for guiding bleeding management of the critically ill patient: a systematic review of the literature. Minerva Anestesiol. 2014;80:1320–35. 24518216

[17] Bolliger D, Szlam F, Molinaro RJ, Rahe-Meyer N, Levy JH, Tanaka KA. Finding the optimal concentration range for fibrinogen replacement after severe haemodilution: an in vitro model. Br J Anaesth. 2009;102:793–9. 10.1093/bja/aep098 19420005

[18] Solomon C, Hagl C, Rahe-Meyer N. Time course of haemostatic effects of fibrinogen concentrate administration in aortic surgery. Br J Anaesth. 2013;110:947–56. 10.1093/bja/aes576 PMC3657602 23388508

[19] Grottke O, Levy JH. Prothrombin complex concentrates in trauma and perioperative bleeding. Anesthesiology. 2015;122:923–31. 10.1097/ALN.0000000000000608 25654437

[20] Gorlinger K, Dirkmann D, Hanke AA, Kamler M, Kottenberg E, Thielmann M, et al. First-line therapy with coagulation factor concentrates combined with point-of-care coagulation testing is associated with decreased allogeneic blood transfusion in cardiovascular surgery: a retrospective, single-center cohort study. Anesthesiology. 2011;115:1179–91. 10.1097/ALN.0b013e31823497dd 21970887

[21] Charbit B, Mandelbrot L, Samain E, Baron G, Haddaoui B, Keita H, et al. The decrease of fibrinogen is an early predictor of the severity of postpartum hemorrhage. J Thromb Haemost. 2007;5:266–73. 10.1111/j.1538-7836.2007.02297.x 17087729

